# Breastfeeding, nutrition and type 1 diabetes: a case-control study in Izmir, Turkey

**DOI:** 10.1186/s13006-022-00470-z

**Published:** 2022-05-27

**Authors:** İpek Çiçekli, Raika Durusoy

**Affiliations:** 1 Department of Nutrition and Dietetics, Faculty of Health Sciences, Acibadem University, İstanbul, Turkey; 2 Department of Public Health, Faculty of Medicine, Ege University, İzmir, Turkey

**Keywords:** Diabetes mellitus, Risk factors, Breastfeeding, Infant, Diet, Child

## Abstract

**Background:**

The relationship between infant breastfeeding and type 1 diabetes mellitus (DM) is unclear but it has been suggested that there may be a link between many environmental factors, including dietary antigens affecting diabetes epidemiology.

The main objective of this study is to investigate nutritional risk factors, especially breastfeeding early in life that may be associated with the development of type 1 DM and to determine the relationship these factors have with the disease.

**Methods:**

This research is a case-control study and was carried out in Ege University Children’s Hospital in İzmir, Turkey between 13 January 2020 and 5 March 2020. A total of 246 children aged between 4 and 14 years were included in the study. The case group consisted of patients diagnosed with type 1 DM followed-up by Ege University Children’s Hospital’s Endocrinology Unit and the control group included non-diabetic children attending the same hospital’s General Pediatric Outpatient Clinic. A structured questionnaire was created by the researchers after reviewing the literature related to nutritional and other risk factors for type 1 DM. The questionnaire was administered by interviewing the parents and it was related to the child, mother and family of the child. In this study, breastfeeding duration was defined as the total duration of breastfeeding and exclusive breastfeeding meant that the child received only breast milk from the mother.

**Results:**

The mean age at diagnosis was 6.30 ± 4.03 years for cases and 7.48 ± 2.56 years for controls. We found that each monthly increase in exclusive breastfeeding duration provided a 0.83-fold (95% CI 0.72, 0.96) decrease in the risk of type 1 DM. Introduction of cereals in the diet at the sixth month or earlier was associated with a 2.58-fold (95% CI 1.29, 5.16) increased risk.

**Conclusions:**

Determining the contribution of exclusive breastfeeding to the disease is important in establishing preventive policies. A longer duration of exclusive breastfeeding may be an important role in preventing the disease. This free intervention that truly works will be cost-effective. Future studies are needed to clarify the role of both exclusive and non-exclusive breastfeeding on the development of type 1 DM.

**Supplementary Information:**

The online version contains supplementary material available at 10.1186/s13006-022-00470-z.

## Background

Diabetes mellitus (DM) is a chronic metabolic disease characterized by hyperglycemia due to impairments in either insulin secretion and / or insulin effect [1]. As of today, 537 million people worldwide have diabetes [2]. This number is estimated to reach 643 million in 2030 and 783 million in 2045, which can be considered alarming levels [2].

Type 1 DM is characterized by insulin deficiency and hyperglycemia, usually starting in childhood, when the beta-cells of the pancreas are destroyed by autoimmune or non-autoimmune processes [2]. In individuals with genetic predisposition (human leukocyte antigen or HLA groups at risk), autoimmunity is triggered by the effect of environmental factors (viruses, toxins, emotional stress, others) and progressive beta-cell damage begins. Clinical symptoms of diabetes occur when beta-cell reserves are reduced by 80–90% [3].

It has been suggested that there are many environmental factors, including dietary antigens [4–6], as well as genetic risk factors [7–11] that affect the epidemiology of type 1 DM [12]. Although not all genotypes with risk have yet been identified, only about 10–15% of individuals at genetic risk develop type 1 DM [5]. In studies conducted on migrants, it has been shown that the incidence of type 1 DM increases in those who migrate from a region where the incidence of type 1 DM is low to a region with high incidence, and the effect of environmental conditions has been emphasized [13]. These data were found to be consistent with the results of studies finding that environmental triggers increase and accelerate the development of clinical type 1 DM despite lower genetic predisposition [13].

Some nutritional factors contribute to the development of the disease. Studies in 40 countries worldwide have shown that dietary patterns may impact the development of type 1 DM [14]. Vitamin D, another nutritional factor, may have a protective effect on glycemic control in patients with type 1 DM [15] and according to a birth cohort study, the provision of vitamin D supplementation for infants early in life could help to reduce the risk of the disease [16]. The introduction of cow’s milk-based infant formulas in the first three postnatal months was found to be associated with an increase in pancreatic beta-cell auto-antibodies [17]. However, another study had shown that cow’s milk did not play an important role in the development of type 1 DM [18].

Although many studies have been performed to investigate the role of nutrition in pregnancy and early in life on type 1 DM, the results have been inconsistent. Breastfeeding [19], probiotic supplementation [20], vitamin C, and zinc supplementation [21] have been shown as possible protective factors against type 1 DM whereas early exposure to eggs, gluten [22, 23] and vegetables [24] might increase the risk.

Studies with school-age children have shown that diabetic children are significantly more prone to stress and depression compared to non-diabetic children [25]. Beyond the psychological and somatic effects of the disease on the individuals, diabetic individuals also encounter socio-economic consequences affecting their families and entire societies [26]. Frequent co-morbidities further increase negative socioeconomic consequences, especially in low- and middle-income countries [26].

According to the Social Security Institution’s data in Turkey, the costs of diabetes and its complications amount to approximately 23% of the total health expenditure [27]. In addition, indirect costs such as the loss of productivity of diabetics, the persons caring for the patient and their family are not included in these cost estimates. Therefore the cost does not reflect the psychosocial effects of the losses of quality-adjusted life years. Knowledge of modifiable environmental risk factors in type 1 DM can assist authorities in planning and implementing preventive policies to reduce the burden of the disease. It is as yet uncertain how and which nutritional or other environmental factors are important in the development of type 1 DM. Moreover, epigenetic mechanisms are not clearly defined.

The main objective of this study is to investigate potential nutritional risk factors, especially breastfeeding early in life, that may be associated with the development of type 1 DM and to determine the relationship of these factors with the disease, independent of other established risk factors.

## Methods

### Participants

A case-control study was carried out at Ege University Children’s Hospital, İzmir City, Turkey, over a period of two months from January to March 2020.

A minimum sample size of 105 cases and 105 controls with a total of 210 participants was calculated with G-Power using the t-test group, with an effect size of 0.5, an error margin of 0.05, and a power of 95%. About 20% more sample size was added to account for possible non-response and a total of 246 children (120 cases and 126 controls) were included in the study.

The study data were collected at Ege University Faculty of Medicine Children’s Hospital in Bornova, Izmir between 13 January 2020 and 5 March 2020. Children and their parents who attended the general pediatrics and endocrinology / metabolic diseases outpatient clinics of the hospital and who met the study criteria were examined. The case group consisted of 120 children in the age group of 4–14 years who were diagnosed with type 1 DM based on World Health Organization and International Diabetes Federation guidelines [28] and who were being followed-up at Ege University Children’s Hospital Endocrinology / metabolic diseases outpatient clinic.

The diabetes outpatient clinic is held once a week (on Thursdays) and on the first Monday of every month. The mean number of diabetic patients attending the research was 15 patients per day. The control group comprised 126 non-diabetic children selected from the general pediatric outpatient clinic of the same hospital. A questionnaire was applied face-to-face to the parents of the children. All questions in the study were asked to the parents and separately written informed consent was obtained from children and their parents. In addition, the files of the case group were examined and the date of diagnosis, height, body weight and HbA1c levels at the time of diagnosis were collected as data.

Children who were followed up in the Endocrine and Metabolic Diseases Outpatient Clinic, diagnosed with type 1 DM and aged between 4 and 14 years were included in the case group. Children who attended the General Pediatrics Outpatient Clinic, were not diagnosed with type 1 DM, and aged 4–14 years were included in the control group. Those who did not want to share their information and could not remember answers to the study questions were excluded. The response rates were 96 and 91% among cases and controls, respectively, for all eligible cases and controls attending the hospital.

### Questionnaires

A structured questionnaire was created by the researchers after reviewing the literature related to nutritional and other risk factors for type 1 DM [21, 29–34]. The questionnaire was administered by interviewing the parents and its content was related to the child, mother and family of the child. For children: anthropometric data, breastfeeding duration, infant formula consumption, the introduction of some foods into the diet, infections, supplementations (vitamin D and probiotic) early in life and physical activity were questioned; for mothers, anthropometric data and history during pregnancy; for family, socio-demographic characteristics such as education, whether the child lived with parents, and family history were asked. In addition, the case group was examined about the age at diagnosis of the disease, the HbA1c level and the percentiles at diagnosis.

In this study, breastfeeding duration was defined as the total duration of breastfeeding and exclusive breastfeeding meant that the child received only breast milk (no other liquids or solids given, not even water with the exception of oral rehydration solution, or drops / syrups of vitamins, minerals or medicines) from the mother [35].

The percentiles were calculated based on the percentile values table of Neyzi et al. [36]. Parents’ body mass index (BMI) was classified according to the World Health Organization’s obesity scale [37]. Finally, high-intensity physical activity was defined as “physical activities that increase the maximum heart rate by 70 − 85%” [38]. Examples of physical activities were given (running, basketball, football, tennis, swimming, skipping rope) by the researcher.

### Statistical analysis

The data were analyzed by using SPSS software. The quality of the data had been checked prior to analysis. Descriptive variables of cases and controls were compared with Student t-tests (continuous variables), Mann Whitney U tests (non-parametric) and chi-square tests (categorical variables). In order to reveal the relationship between significant parameters and the development of type 1 DM independently from other factors, age and sex-adjusted logistic regression analysis were performed. Since the difference in mean ages of the two groups was found to be significant (both age of enrolment in the study and age at diagnosis type 1 DM), other variables were evaluated adjusting for age and gender.

General pediatric outpatient clinic admissions are due to newly developing acute conditions and 85–90% are first visits to the hospital. Ten to 15 % are invited for follow-up one month later, so the follow-up is also at the same age. If they also have a chronic condition, they are referred to pediatric specialization clinics and start follow-up in those clinics.

Among the cases, six were diagnosed with type 1 DM at zero years, three of whom were excluded from the multivariate analysis since they were diagnosed in their first month of life, so the diagnosis would be before the environmental exposures could happen. The remaining three children were diagnosed at 10, 11 and 11 months, thus they were kept in the analysis since they could be exposed to potential nutritional risk factors in question.

Sex and age-adjusted multivariable logistic analysis, adjusted odds ratios (aORs) and 95% confidence intervals (95% CIs) were used to identify possible risk factors of the disease. In all analyses, *p* <  0.05 was considered statistically significant. The dependent variable was having type 1 DM. Maternal factors, family history, family characteristics, nutritional characteristics early in life were the independent variables.

Population Attributable Risk (PAR) and Population Attributable Risk Percent (PAR %) were calculated to estimate the proportion of cases for whom the disease is attributable to exclusive breastfeeding and to estimate the excess rate of type 1 DM in the study population of both exposed and non-exposed children that is attributable to being non-breastfed exclusively up to the first six months. This measure was calculated as [39]:

$$\mathrm{PAR}\:=\:\mathrm{Attributable}\;\mathrm{Risk}\;\mathrm{x}\; \mathrm{Proportion}\; \mathrm{of}\; \mathrm{exposed}\; (\mathrm{P}_\mathrm{e})$$

## Results

### Characteristics of children

A total of 246 children were included in the study, with 120 cases and 126 controls. The mean age of the case and control groups was 10.43 ± 3.31 and 7.48 ± 2.56 years, respectively (*p* <  0.05). The cases’ mean age at diagnosis was 6.30 ± 4.03 years and was found to be significantly lower than the control group. The mean duration of their disease was 4.2 ± 3 .85 years. The mean height percentile was higher in controls (means 45.66 ± 31.16 and 58.00 ± 31.88, *p* = 0.003) and the mean BMI percentile was higher in the case group (means 55.20 ± 29.86 and 40.32 ± 35.02, *p* < 0.001). A significant difference was found in the family history of type 1 DM. There was a type 1 DM history in 10.7% of the case group and 0.8% in the control group (*p* = 0.001). No significant difference was found in the child’s living status with parents and parents’ education level. However, a significant difference was found in physical activity levels (*p* = 0.014). There was no difference between the duration of vitamin D use. In both groups, no infant was supplemented with probiotics in the first year postpartum. The controls’ rate of living in urban areas was found to be significantly higher (Table 1).

**Table 1:** The main characteristics of children

	Cases	Controls	***p***
Sex *(n = 246)*			
*Female*	63 (53.8%)	54 (46.2%)	0.130
*Male*	57 (44.2%)	72 (55.8%)	
Age at enrolment *(n = 246)*	10.43 ± 3.31	7.48 ± 2.56	**< 0.001**
Age at diagnosis (cases)	6.30 ± 4.03	7.48 ± 2.56	**0.006**
Duration of Type 1 DM *(years, n = 120)*	4.16 ± 3.85		
Birth weight *(n = 242)*			
*< 2500 g*	15 (12.5%)	12 (9.8%)	0.515
*2500*–*3999 g*	97 (80.8%)	105 (86.1%)	
> *4000 g*	8 (6.6%)	5 (4.1%)	
Infection in the first year after birth *(n = 241)*			
*Yes*	63 (53.4%)	55 (46.6%)	0.222
*No*	56 (45.5%)	67 (54.5%)	
Diarrhea in the first year after birth *(n = 189)*			
*Yes*	35 (43.2%)	38 (35.1%)	0.262
*No*	46 (57.8%)	70 (65.9%)	
Physical activity *(n = 241)*			
*Any*	66 (55.4%)	89 (73.0%)	**0.014**
*Once or twice a week*	38 (31.9%)	21 (17.2%)	
*Three or more a week*	15 (12.6%)	12 (9.8%)	
Transportation to school *(n = 240)*			
*Does not attend school*	11 (9.1%)	11 (9.1%)	0.604
*On foot*	56 (46.3%)	62 (50.4%)	
*By school bus*	22 (19.0%)	26 (21.5%)	
*By car / bus*	30 (25.6%)	22 (18.2%)	
Residence *(n = 243)*			
*Urban*	34 (28.3%)	54 (43.9%)	**0.011**
*District*	83 (69.1%)	62 (50.4%)	
*Rural*	3 (2.5%)	7 (5.7%)	

### Maternal characteristics

The mean birth interval was higher in the case group. A significant difference was found in the birth intervals with univariate analysis (*p* = 0.036) but not in multivariate analysis. Those with a birth interval of more than six years constituted 20.7% of the cases and 8.0% of controls (Table 2). In the case group, no mother was supplemented with probiotics during pregnancy and 98.4% of the control group were not supplemented with probiotics during pregnancy.

**Table 2:** Maternal factors

	Cases	Controls	***p***
Mean age at birth *(years, n = 246)*	27.48 ± 5.26	27.89 ± 5.41	0.544
BMI classification before pregnancy *(n = 235)*			
*Under weight*	6 (5.3%)	8 (6.6%)	0.444
*Normal weight*	72 (63.7%)	85 (69.7%)	
*Over-weight*	26 (23.0%)	18 (14.8%)	
*Obese*	9 (7.9%)	11 (9.0%)	
Mean of total weight gained during pregnancy *(n = 239)*	14.51 ± 12.53	13.06 ± 8.53	0.295
Oral GTT during pregnancy *(n = 243)*			
*Yes*	83 (70.9%)	96 (76.2%)	0.353
*No*	34 (29.1%)	30 (23.8%)	
Gestational diabetes mellitus during pregnancy* *(n = 188)*			
*Yes*	17 (19.1%)	13 (13.1%)	0.264
*No*	72 (81.9%)	86 (86.9%)	
Form of delivery *(n = 244)*			
*Vaginal*	40 (33.7%)	43 (34.4%)	0.897
*Cesarean section*	79 (66.3%)	82 (65.6%)	
Mean of birth order *(n = 246)*	1.64 ± 0.74	1.68 ± 0.83	0.666
Birth interval *(n = 244)*			
*First child*	49 (41.1%)	54 (43.2%)	**0.043**
*< 3 years*	20 (16.8%)	24 (19.2%)	
*3*–*6 years*	26 (21.8%)	37 (29.6%)	
*> 6 years*	24 (20.1%)	10 (8.0%)	
Supplemented with iron during pregnancy *(n = 246)*			
*Yes*	99 (82.5%)	97 (77.0%)	0.283
*No*	21 (17.5%)	29 (23.0%)	
Supplemented with Vitamin D during pregnancy (*n* = 230)			
*Yes*	62 (59.0%)	75 (60.0%)	0.883
*No*	43 (41.0%)	50 (40.0%)	
Supplemented with probiotic during pregnancy (*n* = 246)			
*Yes*	–	2 (1.6%)	
*No*	120 (100.0%)	124 (98.4%)	

*BMI* Body mass index, *Oral GTT* oral glucose tolerance test

*This question was asked only of those who had an oral GTT during pregnancy, and nine mothers who had DM before pregnancy were also considered to have gestational DM

### Nutritional profiles of children

The mean duration of exclusive breastfeeding was higher in the control group (*p* = 0.009). In the case group, the rate of exclusive breastfeeding for less than one month was 47.8, and 30.6% in the controls (Table 3). This difference was statistically significant (*p* = 0.037). No statistically significant differences were found between colostrum consumption, total breastfeeding duration, infant formula consumption and formula preferences.

**Table 3:** Nutritional characteristics of children early in life

	Cases	Controls	***p***
Received breast milk within first hour after birth (*n* = 243)	102 (85.7%)	102 (82.2%)	0.463
Colostrum-fed (*n* = 243)	114 (95.7%)	111 (89.5%)	0.062
Exclusive breastfeeding duration (n = 239)*			
*Any or less than 1 month*	55 (47.8%)	38 (30.6%)	**0.037**
*1*–*2 months*	18 (15.6%)	28 (22.6%)	
*3*–*5 months*	26 (22.6%)	30 (24.2%)	
*6 months*	16 (13.9%)	28 (22.6%)	
Total breastfeeding duration (n = 241)*			
*< 6 months*	21 (17.9%)	20 (16.1%)	0.977
*6*–*12 months*	27 (23.0%)	28 (22.5%)	
*13*–*24 months*	62 (52.9%)	57 (45.9%)	
*≥ 24 months*	14 (11.9%)	12 (9.6%)	
Introduction of formula (n = 238)*			
*Not consumed*	56 (45.5%)	63 (54.2%)	0.064
*< 6 months*	37 (30.0%)	49 (39.8%)	
*≥ 6 months*	22 (19.1%)	11 (8.9%)	
	**Mean ± SD**	**Mean ± SD**	***p***
Exclusive breastfeeding duration (month)*	1.88 ± 2.23	2.67 ± 2.38	**0.009**
Total breastfeeding duration (month)*	16.04 ± 10.80	16.38 ± 10.08	0.801

*DM* diabetes mellitus, *SD* standard deviation

*Three children who were diagnosed with type 1 DM before the first month after birth were excluded from the analysis

No statistically significant difference was observed between which month the cow’s milk, eggs, fruits, vegetables, and berry fruits were introduced. However the introduction of cereals was statistically significant and the cases’ introduction to them was earlier (*p* = 0.008). For the case group 5.5% were introduced to cereals before the sixth month as compared to 3.2% of controls, while 44.2% of controls were introduced to cereals after the eighth month, compared to 24.7% of cases (Table 4).

**Table 4:** Complementary feeding and introduction to some foods that may affect the development of type 1 DM^*^

	Cases	Controls	***p***
Introduction of complementary foods (month)	5.91 ± 1.38	6.08 ± 2.06	0.456
Introduction of complementary foods *(n = 240)*			
*< 6 months*	26 (22.2%)	23 (18.6%)	
6 *months*	79 (67.5%)	87 (70.7%)	0.769
*> 6 months*	13 (11.1%)	13 (10.5%)	
Cow’s milk *(n = 232)*			
*< 6 month*	14 (12.3%)	16 (13.4%)	0.921
*Between 7* and *12 months*	72 (63.7%)	77 (64.7%)	
*> 12 months*	27 (23.8%)	26 (21.8%)	
Cereals *(n = 231)*			
*< 6 months*	6 (5.5%)	4 (3.2%)	**0.008**
*Between 6* and *7 months*	76 (69.7%)	64 (52.4%)	
*≥ 8 months*	27 (24.7%)	54 (44.2%)	
Egg *(n = 234)*			
*< 6th month*	5 (4.5%)	8 (6.5%)	0.284
*Between 6* and *7 months*	62 (55.8%)	78 (63.4%)	
*≥ 8th month*	44 (39.6%)	37 (30.1%)	
Fruits and vegetables *(n = 239)*			
*< 6 months*	21 (18.1%)	16 (13.0%)	0.510
*Between 6 and 7 months*	86 (74.1%)	95 (77.2%)	
*≥ 8 months*	9 (7.8%)	12 (9.8%)	
Berry fruits (n = 239)*			
*< 6 months*	19 (16.3%)	16 (13.0%)	0.688
*Between 6* and *7 months*	88 (75.8%)	95 (77.2%)	
*≥ 8 months*	9 (7.7%)	12 (9.8%)	

*DM* diabetes mellitus

*Three children who were diagnosed with type 1 DM before the first month after birth were excluded from the analyses

### Multivariate analysis

**Fig. 1:**
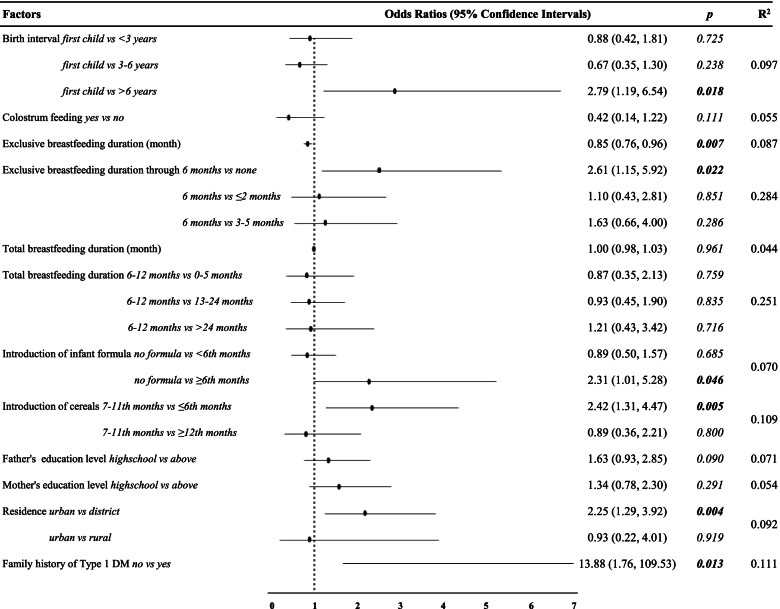
Sex and age adjusted (the cases’ age at diagnosis) logistic regression analysis of risk factors independent of other risk factors

According to non-parametric correlation analyses, exclusive breastfeeding duration and total breastfeeding duration were not found to be associated with age when type 1 DM was diagnosed. The birth interval was found to be significant in the age and sex-adjusted regression analysis. In addition, regardless of age and gender, it was observ ed. that the risk of type 1 DM decreased 0.85 (*p* = 0.007; 95% CI0.76, 0.96) times with each monthly increase in the duration of exclusive breastfeeding (Fig. 1). Having a birth interval of more than six years increased the risk of the disease by 2.79 (*p* = 0.018; 95% CI 1.19, 6.54) times.

According to results of the multivariate logistic regression, longer exclusive breastfeeding duration, living in a rural area and not consuming infant formula were identified as protective factors. Although there was no significant difference found in type 1 DM risk with introduction to cereals at 12 months and after, it was found that the introduction to cereals at the sixth month and earliere increased the risk of type 1 DM by 2.58 (*p* = 0.008; 95% CI 1.29, 5.16) times compared to between months7–11, independent of other risk factors. Similarly, infant formula consumption after the sixth month was associated with an increased risk of type 1 DM compared to no infant formula consumption (Table 5).

**Table 5:** Multivariate logistic regression analysis of selected variables

Factors ***(if categorical, reference categories in superscript)***	***p***	aOR (95% CI)
Age	**0.014**	0.88 (0.80, 0.98)
Sex *girl* vs *boy*	0.105	0.60 (0.32, 1.11)
Birth interval *first child*^*ref*^	0.178	
* < 3 years*	0.869	1.07 (0.46, 2.50)
* 3*–*6 years*	0.317	0.66 (0.29, 1.49)
* > 6 years*	0.106	2.32 (0.84, 6.43)
Exclusive breastfeeding duration (month)	**0.010**	0.83 (0.72, 0.96)
Formula consumption *no formula*^*ref*^	**0.010**	
* < 6 t months*	0.113	0.56 (0.28, 1.15)
* ≥ 6 months*	**0.034**	2.77 (1.08, 7.09)
Introduction to cereals *7–11 months*^*ref*^	**0.006**	
* ≤ 6 months*	**0.008**	2.58 (1.29, 5.16)
* ≥ 12 months*	0.694	0.82 (0.30, 2.22)
Family history of type 1 DM *no* vs *yes*	0.075	6.86 (0.83, 57.09)
Mother’s education level *high school* vs *above*	0.587	1.21 (0.61, 2.37)
Residence *urban*^*ref*^	0.373	
* district*	0.166	1.58 (0.83, 3.03)
* rural*	0.905	1.10 (0.23, 5.37)

### Sensitivity analyses

The potential impacts on our results of age at which the cases developed DM, (whether including or excluding data for the three children who were diagnosed at the first month after birth) and with data missing for the father’s education level variable, was assessed using multiple imputations, as described in the Supplementary Data. Among the cases, six of them were diagnosed with type 1 DM in the first year of life, three of whom were excluded from the multivariate analysis since they were diagnosed in their first month of life, so the diagnosis would have occurred before the environmental exposures could happen. The remaining one child was diagnosed at ten months and two children at 11 months, thus they were kept in the analysis since they could have been exposed to the potential nutritional risk factors in question.

The main analyses were repeated after adjusting for age and the father’s education level. Multiple imputations changed some of the conclusions based on the research sample as attached (Supplementary Table 1). The missing data on the father’s education level in the study was assessed using multiple imputations and this did not change the conclusions. So the model excluding the father’s education level and cases that developed type 1 DM before the first month was used.

PAR was calculated as 0.111 in the study and PAR% was calculated as 38.3% .

## Discussion

Elimination of preventable environmental risk factors associated with type 1 DM is an important step in the prevention of the disease. However, it has not been precisely explained which factors play a key role and when and in which situations the factors should be eliminated [22]. In this research we have explored possible preventable environmental triggers and determinants, especially breastfeeding early in life.

We found that each monthly increase in the duration of exclusive breastfeeding but not total breastfeeding provides a reduction in type 1 DM risk. However, introducing the cereals before the sixth month was found to be an important risk factor. The birth interval which was significant in univariate analyzes, lost its significance in multivariate analysis.

### Breastfeeding

The effect of breast milk, the first food of the newborn, on type 1 DM is a controversial issue. There are many studies in the literature that show no effect [40], a protective effect [19] and an effect [21]. It has been suggested that the protective effect of breastmilk is through reducing neonatal intestinal permeability [41]. The World Health Organization recommends feeding exclusively breast milk in the first six months of life and breastmilk up to the age of two, because feeding children with exclusively breast milk for the first six months after birth prevents diarrhea, respiratory diseases and provides all the nutrients and fluids the infant needs for optimal growth and development [42]. For participants in our study, it was observed that the rates of those who did not receive breast milk at all or those who were exclusively breastfed for less than a month were quite high. It has been observed that the accomplishment rate of the World Health Organization target for six months exclusive breastfeeding is low.

According to the Turkey Demographic and Health Survey data 2018, approximately two in five children were exclusively breastfed up to six months old and the proportion of children who are exclusively breastfed decreases with age; from 59% among 0–1 month-old infants to 14% among 4–5 months old infants [43]. On the other hand, the National Immunization Survey results indicated that only one in four children was breastfed exclusively through six months in the U.S. [44]. In our study, only the median month of breastfeeding was close to the Turkey Demographic and Health Survey data. The exclusive breast milk receiving rates through six months were found to be lower than the worldwide, National Immunization Survey and Turkey Demographic and Health Survey data in both the case and control groups. This was not surprising because our sample was quite low compared to the aforementioned samples, and the data mentioned reflected a population of children younger than two years old in 2018. So the mean age of the children in our study was higher. This result may be different in studies to be conducted with a larger population and adjusted for age. There are large differences in breastfeeding rates between regions, between and within countries. But unfortunately, these rates are insufficient both in the world and in Turkey. We can estimate that 38.3% of type 1 DM cases would be avoided by an increase in the proportion of infants exclusively breastfed to six months. Keep in mind that almost two in five infants who are not breastfed exclusively for the first six months will have type 1 DM so any intervention that can promote breastfeeding may have a big impact in preventing the disease.

In the study of Çarkçı and Altuğ (2020), conducted in the same city as this study (İzmir) the rate of children with type 1 DM who received exclusive breast milk up to the first six months was found to be more than four times compared to our study [45]. While asking the duration of exclusive breastfeeding, the definition of exclusive breastfeeding was explained as “the total time in which the baby takes only breast milk, and no other liquid (including water) or solids other than oral rehydration solution or vitamins, minerals or drugs/syrups are given” in this study. While making a statement, after the parents answered, “Have you ever given water during this period?” was asked again to be sure. In this process, there were parents who changed their answers after the second question. Therefore, different results may have been obtained in studies where this distinction was not made clear.

There are many studies on the relationship between breastfeeding and type 1 DM. Holmberg et al. (2007) found that the duration of total breastfeeding for less than four months is a risk factor for the development of beta-cell autoimmunity in children under five years old. The same study reported that the duration of exclusive breastfeeding for less than four months increased the risk of developing beta-cell autoantibodies two times [17]. In another study, it was shown that the risk of type 1 DM in childhood can be reduced by 15%, even by breastfeeding exclusively in the early weeks of life. However, the observed relationship between exclusive breastfeeding and type 1 DM could not be explained independently of certain risk factors for DM such as gestational DM, birth weight, gestational age, maternal age, birth order and mode of delivery [19]. However in a series of prospective and birth cohort studies investigating the relationship between breastfeeding and the development of islet autoimmunity, no effect of breastfeeding has been reported [46, 47]. Similarly, a series of prospective studies investigating the relationship between breastfeeding and the development of type 1 DM reported that breastfeeding had no effect [48, 49].

We found that longer exclusive breastfeeding duration was a significant protective factor against type 1 DM but the same effect was not observed with total breastfeeding duration. In some studies, exclusive breastfeeding and total breastfeeding duration were not compared and the duration of total or any breastfeeding was researched. Therefore the differences in studies’ results can be attributed to their methods.

In addition to distinguishing between exclusive breastfeeding and total breastfeeding, there are also differences between studies in defining exclusive breastfeeding. For instance, in two Large Scandinavian Birth Cohorts, breastfed infants were found to be at doubled risk of type 1 DM compared to infants who did not receive breast milk at all but no evidence indicated that longer duration of breastfeeding was associated with a reduced risk of the disease [50].

Similarly, in two large cohort studies, breastfeeding duration was not associated with type 1 DM [51, 52]. Infants classified as exclusively breastfed were allowed water-based drinks in the aforementioned study and duration of exclusive breastfeeding was not taken into account.

As can be seen, many studies only look at total breastfeeding duration without making any distinction between exclusive breastfeeding duration and total breastfeeding duration. In addition, striking differences in the breastfeeding practices of governments and health authorities may be a confounding factor in the results of the studies. Positive social norms that support and encourage breastfeeding, including in public spaces, encourage mothers to breastfeed [53]. As observed in our study, the duration of exclusive breastfeeding of children after birth is quite low and it has been observed that parents do not attach sufficient importance to the period of exclusive breastfeeding.

Support from trained counselors and peers, including mothers and other family members, is as important as postpartum health care in maintaining breastfeeding in communities. The support of men, spouses and partners should not be ignored in this process [53]. In studies on breastfeeding, the mother has always been at the center and studies on the role of fathers / partners are insufficient. Tohotoa et al. highlighted the importance of the role of fathers in encouraging and supporting a successful breastfeeding process [54]. Moreover, paternal practical, physical and emotional support could make a difference [54].

When challenges experienced by mothers are shared with their partners, babies might have a better chance of receiving exclusive breast milk for the recommended six months and could keep going on breastfeeding for up to two years. In this way, the early introduction of complementary foods, especially cereals, which we found a significant risk factor for type 1 DM in our study, could be prevented.

### Cow’s milk and infant formula consumption

Early exposure to cow’s milk proteins has been studied in terms of beta-cell autoimmunity and the risks of clinical disease development [55]. Early introduction of cow’s milk proteins into the diet may trigger inflammation of the intestinal mucosa and increase intestinal permeability [56]. The introduction of infant formula reflects the total duration of the exclusive breastfeeding [31]. Therefore, it should be considered together with the duration of exclusive breastfeeding. These may have led to contradictory results [31].

Some studies have shown that early exposure to cow’s milk proteins increases the risk of beta-cell autoimmunity [57] and type 1 DM [58] while others found no relationship between type 1 DM and cow’s milk proteins [31, 59]. We also did not find an association with the timing of cow’s milk introduction. It has been observed that consumption of infant formula at six months and later increased the risk of type 1 DM in this research. However, while the risk of type 1 DM was expected to increase with the consumption of infant formula at six months and earlier compared to those who did not consume it, a statistically significant change was not observed.

This result may be explained in three ways: First, there may be a bias in choosing the control group from the same tertiary care and university hospitals with type 1 DM patients. Considering the socioeconomic status of children attending a university hospital, infant formula may have been introduced earlier than the general community and might not represent the healthy control group clearly. Second, there may have been a response bias. Third, since the mean age of children with type 1 DM is significantly higher, parents may have recall bias. Although it is easy to identify potential sources of bias, it is not possible to predict the true impact of these biases on results.

### Introduction to cereals

Gluten, a protein found in barley, wheat and rye has been hypothesized to be one of the nutritional risk factors related to the development of type 1 DM [60]. A study in non-obese diabetic rats concluded that the intra-epithelial infiltration of T cells, the incidence of autoimmune type 1 DM and enteropathies decreased with a gluten-free diet compared to the controls [61]. Introduction of gluten before four months of age was associated with an increased risk of type 1 DM in another study [62]. These results were explained by the hypothesis that the gluten-free diet may prevent gliadin peptides from crossing the intestinal barrier by reducing intestinal permeability, thus preventing the development of pancreatic autoimmunity [63]. Our study supports these arguments since introducing cereals before the sixth month was found to be an important risk factor.

However in our study, the cereals were not questioned for their gluten content, they were questioned overall, but wheat production and consumption ranks in the first place among cereals in Turkey [64]. So wheat-containing cereals (including gluten) are expected to be added into the diet of infants in the transition to complementary foods at first. Nevertheless, it could not be confirmed specifically that gluten exposure was earlier in cases, but it was found that cereal introduction prior to the sixth month was associated with an increased risk of type 1 DM.

## Limitations

We have many limitations in the study. As in our study, case-control studies always have the potential for bias. It is not easy to collect accurate and unbiased data on past exposures. Therefore case-control studies are prone to some sources of bias like recall bias or the control group’s selection from the hospital. Many of the established risk factors were questioned, in order to overcome confounding. However, the gestation week was not questioned at birth, so it could not be evaluated whether the birth weight was normal for gestational age. It was questioned whether they drank water during exclusive breastfeeding, but we did not collect data on when they started to consume water. Therefore this variable maybe could provide a better comparison for exclusive breastfeeding duration in future studies. In addition, the vaccination status of the children was not asked and abortion was not researched while questioning the birth interval and birth order so they may be confounding factors. Since infections in the first three years were questioned by anamnesis, their bacterial / viral status could not be determined and their relationship with enteroviruses could not be investigated.

## Conclusions

Longer exclusive breastfeeding duration may prevent the early introduction of certain nutrients in the diet. Determining the contribution of exclusive breastfeeding and its interactions with protective factors to the disease is important in establishing preventive policies. Breastfeeding is cost-effective and may be a free intervention for the prevention of type 1 DM. Support from partners is a key factor in maintaining breastfeeding in communities. Considering the limitations of the study, systematic reviews with meta-analysis are needed in determining the role of both exclusive and non-exclusive breastfeeding on the development of type 1 DM.

## Supplementary Information

**Additional file 1.**

## Data Availability

The dataset could be obtained from the corresponding author upon reasonable request.

## Abbreviations

HLA: Human leukocyte antigen
PAR: Population attributable risk
PAR %: Population attributable risk percent
