# Supplementary material for: Breastfeeding, nutrition and type 1 diabetes: a case-control study in Izmir, Turkey
Source: Int Breastfeed J. 2022 May 27;17:42. doi: 10.1186/s13006-022-00470-z (PMC9145075; doi:10.1186/s13006-022-00470-z)
Supplement: Supplementary file 1 — Additional file 1. [file 13006_2022_470_MOESM1_ESM.docx]

**Supplementary Table 1** Sensitivity analysis with multiple imputations for age and father’s education levels

|  | **a** | | **b** | | **c** | | **d** | |
| --- | --- | --- | --- | --- | --- | --- | --- | --- |
|  | **N** | **Percent** | **N** | **Percent** | **N** | **Percent** | **N** | **Percent** |
| Included in analysis | 224 | 91.1 | 206 | 83.7 | 206 | 83.7 | 224 | 91.1 |
| Missing | 22 | 8.9 | 40 | 16.3 | 40 | 16.3 | 22 | 8.9 |
| Total | 246 | 100.0 | 246 | 100.0 | 246 | 100.0 | 246 | 100.0 |
| Nagelkerke R Square | 0.289 | | 0.336 | | 0.473 | | 0.417 | |
| **Factors** | ***p*** | **aOR (95% CI)** | ***p*** | **aOR (95% CI)** | ***p*** | **aOR (95% CI)** | ***p*** | **aOR (95% CI)** |
| Age | **0.014** | 0.88 (0.80, 0.98) | **0.010** | 0.87 (0.78, 0.97) | **< 0.001** | 1.43 (1.26, 1.63) | **< 0.001** | 1.38 (1.23, 1.55) |
| Sex *girl^ref^ vs boy* | 0.105 | 0.60 (0.32, 1.11) | 0.155 | 0.61 (0.31, 1.20) | 0.455 | 0.76 (0.36, 1.58) | 0.269 | 0.68 (0.35, 1.34) |
| Birth interval *first child^ref^* | 0.178 |  | 0.066 |  | 0.277 |  | 0.417 |  |
| *Birth interval < 3 years* | 0.869 | 1.07 (0.46, 2.50) | 0.893 | 0.94 (0.37, 2.41) | 0.300 | 1.74 (0.61, 4.93) | 0.310 | 1.61 (0.64, 4.07) |
| *Birth interval 3*−*6 years* | 0.317 | 0.66 (0.29, 1.49) | 0.247 | 0.59 (0.25, 1.44) | 0.951 | 0.97 (0.37, 2.56) | 0.763 | 0.87 (0.36, 2.11) |
| *Birth interval > 6 years* | 0.106 | 2.32 (0.84, 6.43) | **0.045** | 3.04 (1.03, 9.00) | 0.099 | 2.77 (0.83, 9.27) | 0.250 | 1.92 (0.63, 5.82) |
| Exclusive breastfeeding duration (month) | **0.010** | 0.83 (0.72, 0.96) | **0.007** | 0.81 (0.70, 0.95) | **0.006** | 0.79 (0.67, 0.93) | **0.020** | 0.84 (0.72, 0.97) |
| Infant formula consumption *no formula^ref^* | **0.010** |  | **0.003** |  | **0.024** |  | 0.054 |  |
| *< 6t months* | 0.113 | 0.56 (0.28, 1.15) | **0.047** | 0.46 (0.21, 0.99) | 0.341 | 0.67 (0.29, 1.53) | 0.387 | 0.71 (0.33, 1.54) |
| *≥ 6 months* | **0.034** | 2.77 (1.08, 7.09) | **0.025** | 3.22 (1.16, 8.96) | **0.023** | 3.76 (1.20, 11.82) | **0.047** | 2.84 (1.01, 7.95) |
| Introduction to cereal *7*−*11 months^ref^* | **0.006** |  | **0.022** |  | **0.002** |  | **0.004** |  |
| *≤ 6h months* | **0.008** | 2.58 (1.29, 5.16) | **0.013** | 2.64 (1.23, 5.67) | **0.003** | 3.53 (1.52, 8.23) | **0.010** | 2.68 (1.27, 5.66) |
| *≥ 12 months* | 0.694 | 0.82 (0.30, 2.22) | 0.994 | 1.00 (0.34, 2.97) | 0.614 | 0.73 (0.22, 2.48) | 0.424 | 0.64 (0.21, 1.92) |
| Family history of type 1 DM *no^ref^ vs yes* | 0.075 | 6.86 (0.83, 57.09) | 0.125 | 5.44 (0.63, 47.22) | 0.065 | 9.38 (0.87, 101.53) | 0.068 | 8.24 (0.86, 79.12) |
| Mother's education level *highschool^ref^ vs above* | 0.587 | 1.21 (0.61, 2.37) | 0.481 | 1.33 (0.60, 2.92) | 0.436 | 0.71 (0.29, 1.70) | 0.310 | 0.68 (0.33, 1.42) |
| Residence *urban^ref^* | 0.373 |  | 0.128 |  | **0.036** |  | 0.143 |  |
| *district* | 0.166 | 1.58 (0.83, 3.03) | 0.054 | 1.98 (0.99, 3.96) | 0.146 | 1.75 (0.82, 3.73) | 0.335 | 1.41 (0.70, 2.85) |
| *rural* | 0.905 | 1.10 (0.23, 5.37) | 0.902 | 0.89 (0.14, 5.66) | 0.099 | 0.20 (0.03, 1.36) | 0.167 | 0.31 (0.06, 1.63) |
| Father's education level *highschool^ref^ vs above* |  | | 0.889 | 1.06 (0.48, 2.31) | 0.816 | 0.90 (0.38, 2.14) |  | |
|  |  |  |  |  |  |  |  |  |
| a:The age that the cases developed DM except the cases' diagnosis age before the first month and excluding father's education variable from analysis. | | | | | | | |  |
| b: The age that the cases developed DM |  |  |  |  |  |  |  |  |
| c: The age of the cases enrollment in the study |  |  |  |  |  |  |  |  |
| d: The age of the cases enrollment in the study and excluding father's education variable from analysis. | | | | |  |  |  |  |
|  |  |  |  |  |  |  |  |  |
| aOR: Adjusted Odds Ratios |  |  |  |  |  |  |  |  |
| 95% CI: 95% Confidence Intervals |  |  |  |  |  |  |  |  |
